# Cannabidiol from *Cannabis sativa* L. Herbal Extract as an Bioactive Factor in Polysaccharide Coatings with Antioxidant Properties for Extended Food Quality

**DOI:** 10.3390/ma18174081

**Published:** 2025-08-31

**Authors:** Renata Dobrucka, Mikołaj Pawlik, Marcin Szymański

**Affiliations:** 1 Department of Non Food Products Quality and Packaging Development, Institute of Quality Science, Poznan University of Economics and Business, al. Niepodległości 10, 61-875 Poznan, Poland; mikoajpawlik5@gmail.com; 2 Center for Advanced Technologies, Adam Mickiewicz University in Poznan, ul. Uniwersytetu Poznańskiego 10, 61-614 Poznan, Poland

**Keywords:** *Cannabis sativa* L. herb, antioxidant pectin films, active component

## Abstract

In the present study, pectin films with antioxidant activity were obtained in response to the increasing demand for active packaging systems related to the need for safe finished products with a long shelf life. To obtain the films, *Cannabis sativa* L. herb extract was used as the active agent. The samples with the highest extract contents of 20:80 2.0F and 80:20 2.0F (PA:PC ratio + wt.% extract) were characterized by a polyphenols content of 0.9067 ± 0.0184 [%]. They also showed the highest antioxidant activity, at ABTS = 1.79 ± 0.04 [mg/mL] and DPPH = 4.44 ± 0.10 [mg/mL]. Mechanical tests conducted showed that samples without extract addition, regardless of the pectin apple to citrus ratio, were characterized by similar values of mechanical parameters (*p* > 0.05). Spearman’s rank correlation coefficients were used to demonstrate the strength and direction of the relationship between pairs of variables. Statistical analysis showed strong correlations between antioxidant indices and polyphenol content.

## 1. Introduction

Cannabis is among the earliest plants cultivated by humans for both textile production and medicinal purposes. The first information about their medicinal properties comes from China and dates back to 2700 BC [1]. The genus Cannabis includes three species of cannabis, *C. sativa* ssp. sativa (L.), *C. sativa* ssp. indica (Lam.) and *C. sativa* ssp. ruderalis (Janisch), the first being the most common [2]. *Cannabis sativa* L. is an annual plant in the Cannabaceae family, mostly dioecious and sometimes monoecious [3]. Depending on its use and cannabinoid content, cannabis is classified as fibrous (hemp or industrial hemp) or narcotic (medicinal hemp or marijuana) [4]. The domesticated variety *C. sativa* L. is widely cultivated around the world, including in the United States (US), Africa, Europe and Canada. It is a versatile crop with low environmental impact that can be used in various industries, i.e., agriculture, food production, construction, cosmetics and pharmaceuticals. According to the Food and Agriculture Organization (FAO) Stat 2018 report, by area, the largest producers are Canada (555,853 ha), France (12,900 ha) and North Korea (21,247 ha) [5]. Around 70 different types of cannabis are currently regulated for commercial production and distribution in the European Union [6]. More than 50 countries around the world, along with various US states, have taken progressive steps to legalize the cultivation, processing and use of cannabis for recreational and/or medicinal purposes [7].

In the rapidly expanding food industry, packaging plays a pivotal role in maintaining product preservation, safety, convenience, and sustainability. In recent years, the development of innovative solutions—particularly active packaging—has accelerated, driven by several interrelated factors. Among the most significant are increasing consumer expectations for safer, more sustainable, and minimally processed foods with extended shelf life, alongside a rising demand for convenient, eco-friendly, and cost-effective bio-based packaging alternatives [8]. Advances in packaging materials thus present considerable potential to simultaneously address the dual challenges of food preservation and environmental sustainability within the food sector [9].

Researchers are investigating the possibility of producing biodegradable packaging from biomass materials such as polysaccharides and proteins [10]. An example is given by Cao et al. [11], who obtained a packaging film with antioxidant properties by adding soluble soy polysaccharide (SSPS) and pomelo peel extract (PPE). The resulting films exhibited antioxidant properties and improved mechanical properties, enabling lipid preservation. Shah et al. [12] produced composite films by introducing frankincense oleoresin (FOR) into pectin and sodium alginate matrices. Another example is research in which apple pectin, gum tragacanth, and extracts from cherry wine production pomace were incorporated as biocomponents in antioxidant films [13]. In addition, citrus pectin and curdlan gum with the addition of *Rumex hydrolapathum* extract were used to develop active films against *E. coli* [14]. With an increase in the concentration of the extract in the tested film, an increase in the effectiveness of inhibiting the growth of this microorganism was observed. A composite film formulated from apple pectin and citrus pectin in an 80:20 ratio, enriched with an extract derived from unshelled seeds of *Cannabis sativa* L., was evaluated for its antimicrobial properties. The results demonstrated that the prepared films exhibited inhibitory activity against all tested microorganisms. In particular, all film variants showed antibacterial activity against *Salmonella typhimurium* and *Listeria monocytogenes* [15].

For the development of materials for sustainable food packaging, apple and citrus pectin were used in different ratios: 80:20 and 20:80 [16]. In fact, pectins are polysaccharides found as central lamellae in the cell walls and intercellular spaces of plants [17]. For some time, there have been reports of its use in the creation of pectin packaging materials due to its biocompatibility, high availability, low cost and low toxicity [18,19,20,21]. Pectin market, which was valued at USD 1.2 billion in 2022, is projected to grow to reach USD 1.9 billion by 2030 [22]. Tragacanth gum, which is also a product of natural origin, has also been used. Its main source is the resin of the tragacanth plant (*Astragalus gummifer*), which grows mainly in areas of the Middle East [23]. Both pectin and astragalus guramine (E413) have been classified by the World Health Organization/Food and Agriculture Organization (WHO/FAO) Expert Committee as food additives, with no limitation on the level of consumption, demonstrating safety to the consumer and packaged food.

**Figure 1:**
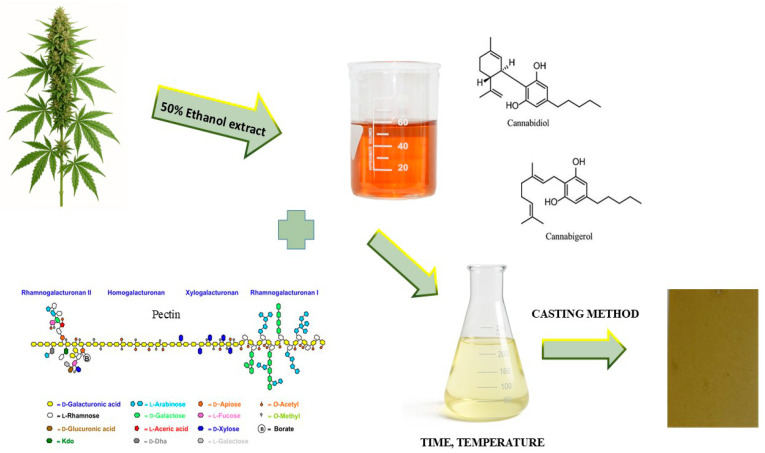
Scheme for obtaining a pectin antioxidant film with the addition of *Cannabis sativa* L. herbal extract.

Natural antioxidants can be directly added to polymer matrices as free radical scavengers or as protectants against autooxidation [24]. Incorporating plant extracts into biodegradable food packaging materials has attracted considerable attention, owing both to their natural origin—which aligns with consumer demand for alternatives to synthetic additives—and to their richness in antioxidants derived from polyphenols and other bioactive compounds [25]. Nowadays, consumers prefer foods that are convenient, of good quality, with a long shelf life, with minimal preservatives and without synthetic additives [26], so plant extracts are widely used. In this study, *Cannabis sativa* L. herbal extract was added to polysaccharide matrices made of apple pectin (PA) combined with citrus pectin (PC) in the following proportions: 20:80 and 80:20. These are pioneering studies, as there are no studies presenting the combination of pectin, tragacanth gum, and extract from the *Cannabis sativa* herb. The obtained films were assessed in terms of the mechanical properties tensile strength (TS) and elongation at break (EB), and barrier properties (WVTR). The color and optical properties of the produced polysaccharide matrices were analyzed, and scanning electron microscopy was also used. A determination of the total polyphenol content (TPC) and an antioxidant study of the pectin films were also performed. Figure 1 shows a scheme for obtaining a pectin antioxidant film with the addition of *Cannabis sativa* L. herbal extract.

## 2. Materials and Methods

### 2.1. Preparation of the Cannabis Herb Extract

Powdered cannabis plant herb was used to prepare the extract. The raw material for the tests consisted of 100% chopped leaves obtained from NatVita (Długołęka, Poland), with the product code 5902096508086. In a round-bottomed flask, 50 g of the herb was placed in a flask, to which 50% ethanol:water (*v*/*v*) was added and kept at the boiling point of the solvent under a reflux condenser for about 1.5 h. The resulting extract was filtered and returned for a second extraction under the same conditions. The resulting filtrates were combined, and then the ethanol was evaporated to a volume of 50 mL to give a concentration of 50% ethanol extract in water at 1 g/mL.

### 2.2. Preparation of Antioxidant Pectin Films

In the next step, the preparation of matrices for the production of antioxidant films began. Pectin apple (PA) (BATOM, Kraków, Poland) was combined with pectin citrus (PC) (BATOM, Kraków, Poland) in the following ratios: 20:80 and 80:20. The prepared mixtures of apple and citrus pectin (PA:PC 20:80 or PA:PC 80:20) were mixed with tragacanth gum (TG) ( Sigma-Aldrich, Munich, Germany) and glycerin (POCH S.A., Gliwice, Poland) in a ratio of 1:1:0.1.

The extract of the herb was added to the solutions in appropriate amounts: 0.5 [wt.%] and 2.0 [wt.%]. The obtained solutions were subject to magnetic stirring at 900 rpm for 65 min at 115 °C. Produced by the cast method, the film samples were dried at 23 °C for 48 h in order to produce a thin film. The obtained foil samples were marked with the letter F (film). The resulting samples were determined as follows: (PA:PC:TG) 20:80 °F, (PA:PC:TG: 0.5 [wt%]) 20:80 0.5F, (PA:PC:TG: 2.0 [wt%]) 20:80 2.0F and (PA:PC:TG) 80:20 °F, (PA:PC:TG: 0.5 [wt%]) 80:20 0.5F, PA:PC:TG: 2.0 [wt%]) 80:20 2.0F.

### 2.3. Study of the Prepared Extract

Analysis was performed on a Bruker gas chromatograph (Bruker Corporation, Massachusetts, United States) with mass detection. A 1.0 g portion of *Cannabis sativa* herb was accurately weighed into a 25 mL round-bottom flask, to which 4 mL of ethanol was added. The mixture was refluxed at the boiling point for 10 min using a heating mantle, subsequently cooled to ambient temperature, and filtered through a 0.45 µm syringe filter. The filtrate was directly subjected to GC–MS analysis.

Gas chromatography–mass spectrometry was performed using a Bruker instrument equipped with electron impact ionization (EI) at 70 eV and an ion source temperature of 200 °C. Helium was employed as the carrier gas at a constant flow rate of 1.0 mL min^−1^. The oven temperature program was as follows: the initial temperature was 60.0 °C (which was held for 3.0 min), and this was ramped to up 280.0 °C at 10.0 °C min^−1^, with a final hold duration of 35.0 min, yielding a total run time of 60.0 min. The coolant temperature was set at 50.0 °C with a timeout of 20.00 min and a stabilization time of 0.50 min.

Compound identification was achieved by comparing retention times and mass spectral data with those in the NIST library, ensuring both spectral match quality and retention index consistency.

Also, the extracts were subjected to TPC evaluation and antioxidation tests, similarly to the previous study [27,28]. The result of the determination is the average of five independent determinations.

### 2.4. Research of Prepared Films

All films produced were stored at a relative humidity of 50 ± 5% and a temperature of 22 °C. The resulting films were subjected to tests including Fourier transform infrared (FTIR) spectroscopy, determination of total polyphenol content (TPC), determination of 2,2 Diphenyl 1 picrylhydrazyl (DPPH) content, determination of 2,2′-azino-bis (3-ethylbenzothiazoline-6-sulfonic acid) (ABTS) content, mechanical testing on a Zwick machine model BDO-FBO 0.5^TH^ (ZwickRoell GmbH, Ulm, Germany), water vapor transmission rate (WVTR) determination, optical testing on a Eurotom Byk haze garde I machine (BYK-Gardner, Geretsried, Germany), according to ASTM D1003-13, color testing using an EnviSense NR60CP colourimeter (Shenzhen ThreeNH Technology Co., Shenzhen, China), the color system recommended by the Comission Internationale de l’Eclairage (CIE), microscopic testing (using an Evo 40 scanning electron microscope (Zeiss, Oberkochen, Germany) and an investigation using a Zeiss SteREO Discovery V8stereo microscope (Carl Zeiss Microscopy Deutschland GmbH, Oberkochen, Germany). In this study, the test parameters and conditions were the same as those used in the previous papers [29,30]. The result of the determination is the average of five independent determinations.

### 2.5. Statistical Analyses

Statistical significance was evaluated at a 95% confidence level. Antioxidant activity (DPPH, ABTS) was calculated using the least squares method. Calibration curves (y = ax + b) were fitted to determine the slope (a), intercept (b), correlation coefficient (R^2^), and standard deviation. Measurement accuracy was assessed using the coefficient of variation (CV). Statistical analyses were performed with EXCEL 2019 and STATISTICA 13.

## 3. Results and Discussion

### 3.1. FTIR Analysis of Pectin Films

**Figure 2:**
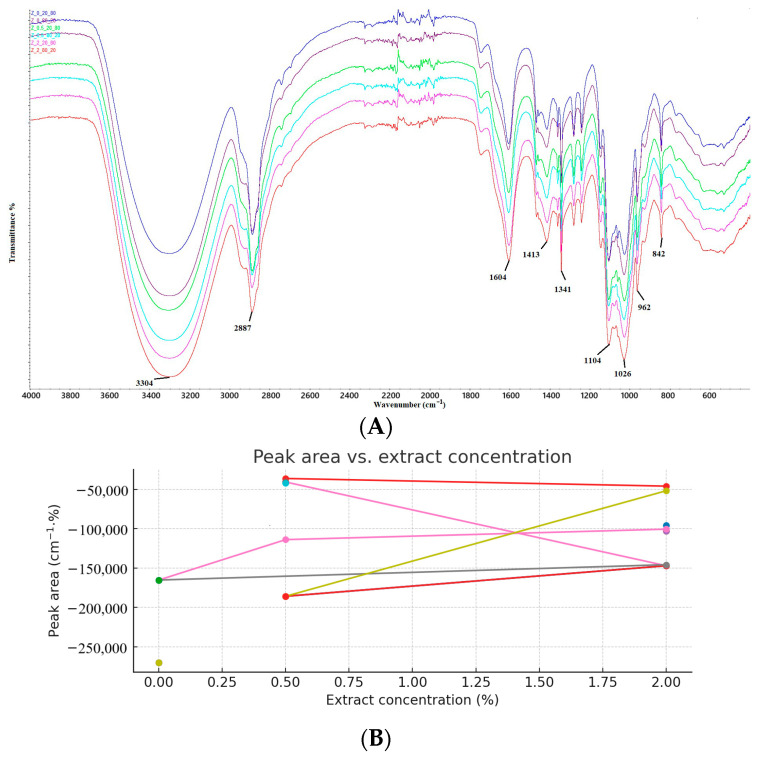
(**A**) FTIR spectra of antioxidant pectin films; (**B**) area vs. extract concentration chart.

FTIR spectroscopy is a relevant method for determining the interactions between the functional groups of the components of the polymers used [31]. In the spectrum (Figure 2A) obtained for the obtained antioxidant pectin films, distinct peaks are present: 3304 cm^−1^, 2887 cm^−1^, 1604 cm^−1^, 1413 cm^−1^, 1341 cm^−1^, 1104 cm^−1^, 1016 cm^−1^, 962 cm^−1^, 842 cm^−1^. The broad peak at 3304 cm^−1^ found in all films is related to the OH vibrations of water molecules in the film structure [32,33]. The sharp peak at 2887 cm^−1^ corresponds to the stretching vibrations of the C-H bonds of the methyl and methylene groups in the produced antioxidant pectin films [34]. The arm which appeared at 1604 cm^−1^ is related to vibrations associated with esterified carboxyl (-COOR) and free carboxyl (-COOH) [30,35,36]. The peaks recorded at 1104 cm^−1^ and 1413 cm^−1^ belong to the C-O stretching oscillations [37]. The sharp peaks at 1104 cm^−1^, 1016 cm^−1^, 962 cm^−1^ and 842 cm^−1^ were attributed to the furanose structure and α- and β-pyranose rings in pectin [38]. Figure 2 shows differences in intensity and peak shape depending on the amount of extract used. These changes provide additional evidence suggesting potential interactions between the active compounds in the extract and the pectin film matrix [39]. Figure 2B shows the relationship between the peak area in the FTIR spectrum and the extract concentration (%), separately for several characteristic absorption bands (marked in the legend in cm^−1^). The legend on the right shows the wavenumber values that correspond to different characteristic vibrations in the tested material. For some bands (e.g., 993 cm^−1^, 1346 cm^−1^), there is a clear increase in the peak area with increasing extract concentration. This may indicate the intensification of a given vibration group in the presence of a larger amount of extract. Other bands (e.g., 1597 cm^−1^, 3921 cm^−1^) show the opposite trend—the surface area decreases with increasing concentration, which may indicate a weakening of a given type of vibration. For several bands (e.g., 824 cm^−1^, 1015 cm^−1^), the changes are non-linear, which may indicate a more complex interaction between the extract components and the sample matrix. The spread of values for individual bands is large, suggesting that the effect of extract concentration is selective and dependent on the specific mode of molecular vibration.

### 3.2. GC-MS Analysis of Cannabis Herb Extract

**Figure 3:**
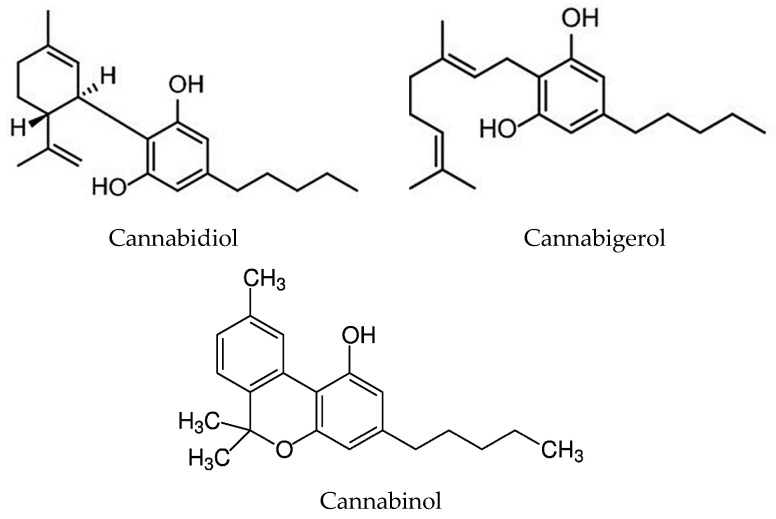
The structure of the selected compounds present in cannabis.

GCMS analysis of the ethanolic extract indicated the presence of a number of compounds of cannabis tea extract (Table 1). Cannabidiol (C_21_H_30_O) was present at a concentration of 80.78 [%] and cannabigerol (C_21_H_32_O_2_) at a concentration of 7.61%. Almost 600 compounds found in *Cannabis sativa* were isolated. Of the most interesting groups of compounds were phytocannabinoids, of which more than 100 were identified. These are members of the terpenophenol group with a characteristic C-21 backbone [40]. Cannabinoids, as secondary metabolites, provide plants with the ability to interact with the environment and are involved in their survival. The predominant form of cannabinoids is carboxylic acid. Decarboxylation of cannabidiolic acid (CBDA) is the process by which cannabidiol (CBD) is obtained [41]. Figure 3 presents the structure of the selected compounds present in cannabis. One of the most important phytocannabinoids is cannabidiol’s companion cannabidiolic acid (CBDA). This acid, together with CBD, is the main component of the glandular hairs of hemp (existing in amounts of up to 15%). In fresh plant material, 95% of CBD is present in acidic form [35]. Additionally, more than 120 phytocannabinoids have been reported in hemp, of which cannabidiol (CBD), Δ9-tetrahydrocannabinol (THC), cannabigerol (CBG) and cannabinol (CBN) are the most common [7].

CBD is the most promising non-psychoactive therapeutic cannabinoid due to its strong antioxidant effects [36]. It has analgesic, anti-inflammatory, anticonvulsant, antiemetic and antipsychotic effects (reducing the negative psychoactive effects of THC) [37]. There is another cannabinoid present in cannabis: cannabigerol (CBG). This chemical has no psychoactive properties [1,42]. CBG has also been identified as a potent anti-inflammatory [43], and CBN is a constituent formed by the non-enzymatic oxidation of the by-product ∆9-THC, after prolonged storage, generally at elevated temperatures [44,45]. Additionally, CBN was the main cannabinoid isolated and identified from “charas”, a resin secreted from cannabis. CBN has been reported in the literature to exhibit a variety of pharmacological properties, including anticancer, antimicrobial, analgesic and anti-inflammatory effects [46].

### 3.3. Selected Properties of Antioxidant Pectin Films (Density, Moisture Content Swelling Index)

**Figure 4:**
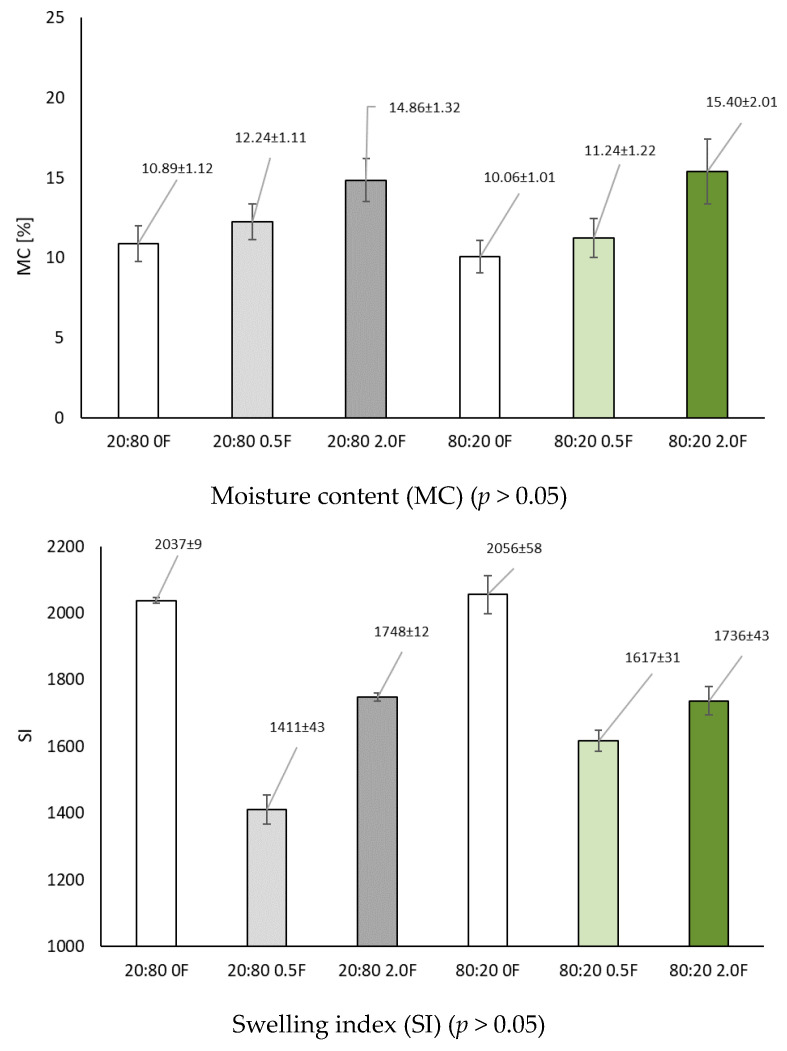
Moisture content (MC) and swelling index (SI) of pectin films.

In this study, film density and swelling index were determined as fundamental factors that determine the ability of the resulting films to retain water [26] (Table 2). Density, on the other hand, is essential to understand the relationship between molecular structure and physical properties such as elastic modulus and barrier properties [47,48]. The addition of the extract to the film matrix resulted in an increase in film density. The highest density values were obtained for films containing the extract, i.e., 20:80 0.5F, 20:80 2.0F, 80:20 0.5F and 80:20 2.0F. The incorporation of the extract increased the density of the films, which directly affected their physical properties. It also led to a higher moisture content (MC) in the polymer matrix (Figure 4). In contrast, the addition of the extract significantly reduced the swelling ratio, with a stronger decrease observed for the 20:80 0.5F and 80:20 0.5F samples. This reduction can be attributed to the formation of strong hydrogen bonds between the polymer blend chains and the polyphenols present in the plant extract [49]. Similar results were reported for the PVA/agar/carrageenan system [50].

### 3.4. Total Polyphenol Content (TPC) and Antioxidant Study of Pectin Films

**Figure 5:**
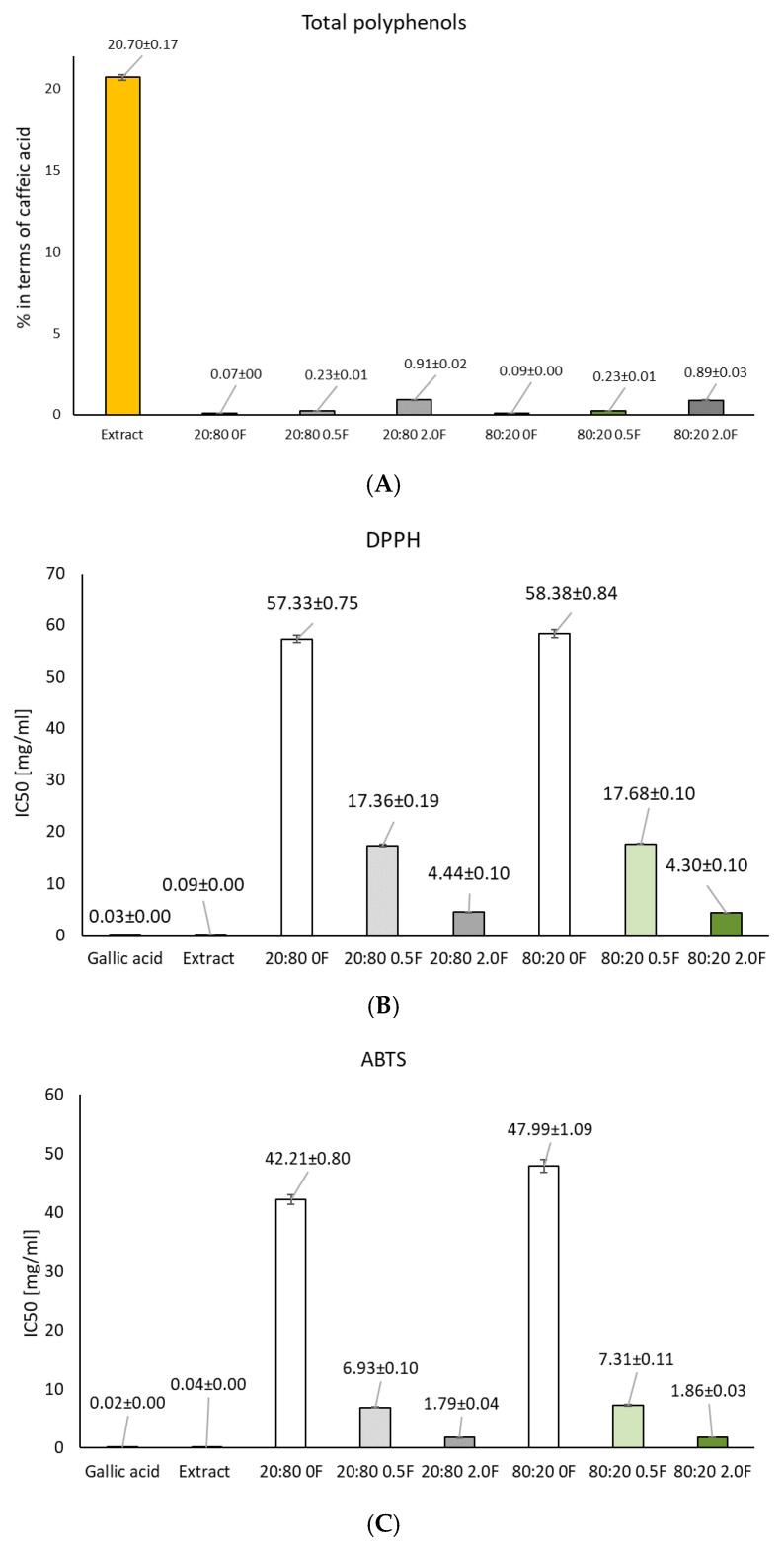
Graphs showing the total polyphenol content (TPC) (**A**) and antioxidant activity (DPPH (**B**); ABTS (**C**)) in the 50% alcoholic extract of cannabis herb and in the films (*p* > 0.05).

In general, oxidation reactions can cause a loss of nutrients and a decrease in the quality of packaged food. Therefore, antioxidant food packaging materials can help reduce the rate of oxidation and extend the shelf life of food [51]. Antioxidant agents in packaging materials can capture free radicals and chelate metal ions to delay oxidation and extend food shelf life. Natural extracts are used in many scientific studies as antimicrobial and antioxidant substances aimed at improving food safety. Natural antioxidants are generally considered safer and may exert additional biological effects owing to the presence of diverse active constituents [29]. Plant extracts represent complex synergistic mixtures of bioactive compounds derived from various botanical sources, such as fruits, flowers, seeds, leaves, and roots [52]. Among these, polyphenols constitute a major group characterized by a wide range of biological activities, including antioxidant, anti-inflammatory, and antimicrobial effects [53]. Quantifying their total content in plant extracts provides important insights into the biological potential of such preparations. The herb extract tested showed a high total polyphenol content (TPC) at 20.7 ± 0.2%. The extract also exhibited high antioxidant activity, as assessed by DPPH and ABTS tests, which showed free radical scavenging activities of 0.0341 ± 0.001 and 0.017 ± 0.003 mg/mL, respectively. In addition. the activity of the extract was slightly weaker than that of gallic acid, which served as a reference in the study. The use of cannabis herb extract in film production allowed the material to be enriched with active polyphenols. Analysis of the total phenolic content (TPC) of the films showed that increasing the concentration of the extract in the films led to a corresponding increase in polyphenol content. Incorporation of the extract into the polymer matrices led to a significant increase in both the total polyphenol content (TPC) and antioxidant activity of the films studied (Figure 5). It depended on the amount of extract used. Test films with the highest extract contents of 20:80 2.0F and 80:20 2.0F were characterized by a TPC content of 0.9067 ± 0.0184%. They also showed the highest antioxidant activity of ABTS = 1.79 ± 0.04 and DPPH = 4.44 ± 0.10 mg/mL. The earlier studies using unshelled hemp seed extract [27] also demonstrated the antioxidant activity of the produced pectin films. For the 2.5 [%wt.] films, the IC50 values were 1095 mg/mL (ABTS method) and 878 mg/mL (DPPH method). Furthermore, in this study, the lowest antioxidant activity was observed for films with the lowest extract content. The obtained films complied with the expected antioxidant activity, which is extremely important in the process of food storage because films containing the highest concentration of cannabis herb extract can be effective in inhibiting lipid peroxidation and vitamin oxidation in food products, these being some of the main causes of food degradation during processing and storage. This process involves a chain reaction initiated by the formation of secondary products and free radicals, which enhance lipid autoperoxidation, deteriorate the overall quality of the food—including its flavor, aroma, and nutritional value—and promote the generation of toxic compounds [24].

### 3.5. Mechanical and Barrier Tests on Antioxidant Pectin Films

**Figure 6:**
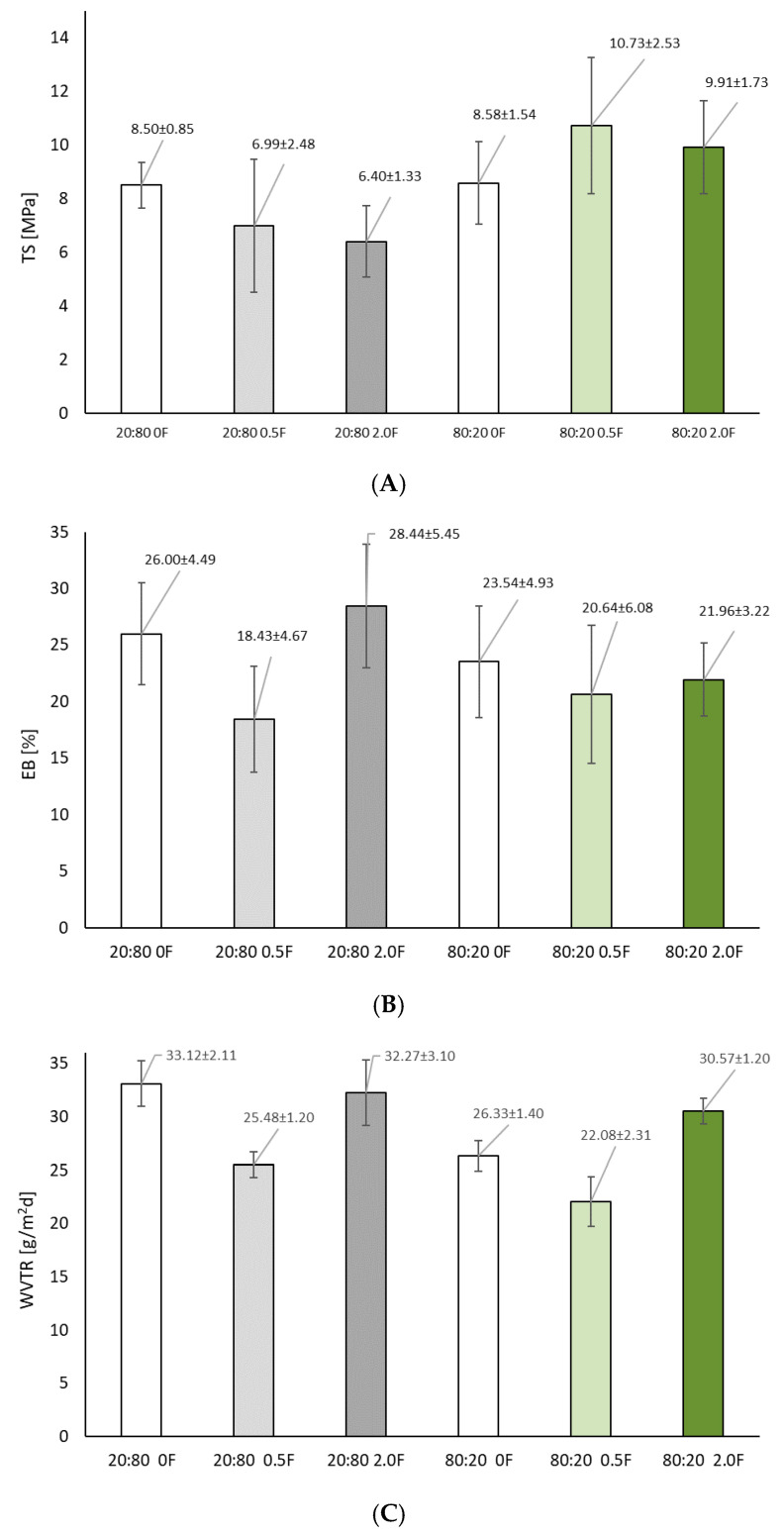
Tensile strength (**A**), elongation at break (**B**) and water vapor transmission test results of antioxidant pectin films (**C**) (*p* > 0.05).

For practical implementations, packaging materials must exhibit appropriate mechanical properties such as tensile strength (TS) and elongation (EB), as well as having adequate barrier properties (WVTR). The lower the water vapor permeability, the higher the performance of the film as a moisture barrier [54]. On the other hand, a higher level of mechanical properties helps preserve food quality during storage and transportation [55]. The mechanical tests carried out showed that zero samples without the addition of extract, regardless of the ratio of PA to PC, were characterized by similar values of the mechanical parameters (TS and EB) (*p* > 0.05) (Figure 6). For the 20:80 °F film, these values were 8.50 ± 0.85 MPa and 26.00 ± 4.49%, respectively. For the 80:20 °F sample, the values were 8.58 ± 1.54 MPa and 23.54 ± 4.93%. The addition of the extract, and thus the amount of polyphenols in the samples, caused an increase in strength and a decrease in elasticity for the 80:20 film. Statistical analysis showed that mechanical properties (TS and EB) are correlated with polyphenol content and antioxidant activity in the samples. The use of the extract resulted in increased film stability due to possible intermolecular bonding between the polysaccharides in the blend, thus forming stronger hydrogen bonds [56]. Increased cross-linking and intermolecular bonding were responsible for the increase in tensile strength [57]. The observed increase in TS for the 80:20 film caused by the addition of the extract can also be attributed to its homogeneous dispersion in the film matrix, which enhanced the film’s cross-linking structure through hydrogen bonding and cross-linking with the molecules of the pectin and gum tragacanth used [58,59], showing that polysaccharides can form complexes with active compounds. These interactions can introduce additional bonds in the film matrix, resulting in a more cohesive but flexible structure, as indicated by our findings. Polyphenols can act as plasticizers and increase the flexibility of active films [60]. However, there are studies that indicate the adverse effects of extracts on films. A study by [61] showed that the tensile strength of Gloiopeltis furcate funoran films with yellow onion peel extract decreased as the concentration of the extract increased. The break values at elongation of the film containing yellow onion peel extract were higher than those of the control film. Another study [62] indicated that with an increase in the basil extract content added to the PVA/starch film, there was a variation in its tensile strength to almost 19.49 MPa. Meanwhile, the WVTR tests carried out in the present study indicated that a lower extract content caused a decrease in the value of this parameter, and thus an increase in the barrier properties of the obtained film (Figure 6). The 20:80 0.5F and 80:20 0.5F films were characterized by WVTR values of 22.08 ± 0.79 g/m^2^ d and 25.48 ± 0.39 g/m^2^ d, respectively. This testified to the best barrier properties of the obtained films. The increased extract concentration could have caused imperceptible phase separation, i.e., small areas rich in CBD could have formed in the matrix that did not fully integrate with the biopolymer matrix. Such microcracks made it so that the diffusion of water vapor more easily occurred. In addition, the larger amount of extract disrupted the crystallization process, increasing the presence of an amorphous structure and, consequently, water vapor transmission.

### 3.6. Optical and Color Tests of Antioxidant Pectin Films

**Figure 7:**
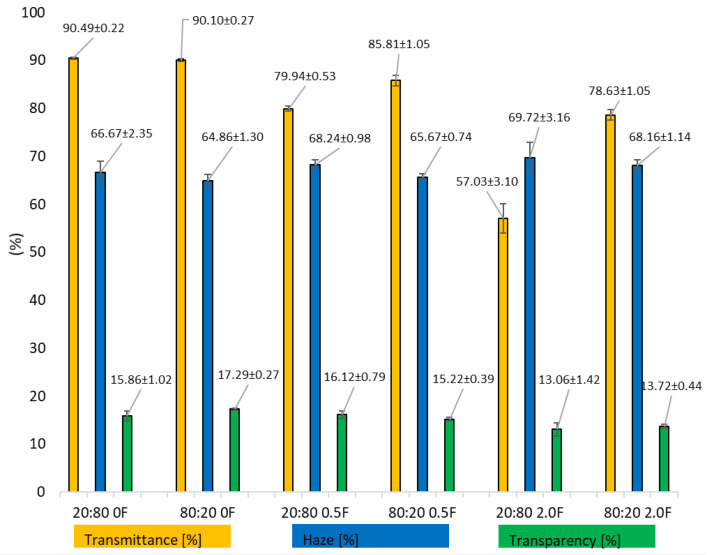
Optical properties (transmittance, haze, and transparency) of the obtained pectin films.

Opacity and transparency are critical properties for membranes intended for food packaging applications. Furthermore, these parameters serve as functional indicators, providing insights into the size of particles dispersed within the polymer solutions [63]. Moreover, color and light transmission are key optical properties of edible films that influence consumer acceptance of food products. Optical characteristics, including UV barrier capacity and transparency, are critical attributes of food packaging films. Efficient UV blocking is particularly important to prevent photocatalytic reactions and light-induced color changes in packaged foods [64]. Figure 7 shows the optical test results of antioxidant pectin films. In the evaluated samples, a clear decrease (*p* < 0.05) in transmittance and transparency was observed for all films after adding the color extract to the matrix. There was, however, an increase in the haze of the samples tested as a function of the amount of extract. The 20:800F and 80:200F films showed haze at H = 66.67 ± 2.35 and H = 64.86 ± 13.00. Through the addition of the maximum amount of the extract, it increased to values of H = 69.72± 3.16 and H = 68.16 ± 1.14. Therefore, the change in the optical parameters of the tested films was clearly related to the addition of the colored extract. The incorporation of polyphenolic extracts into biopolymer films typically increases opacity due to cross-link formation, which alters the refractive index and reduces light transmission [65,66]. This feature is desirable for edible films intended for light-sensitive food products [67,68]. Therefore, it can be concluded that the obtained packaging materials are very beneficial in terms of protecting packaged foods from UV radiation and visible light.

**Figure 8:**
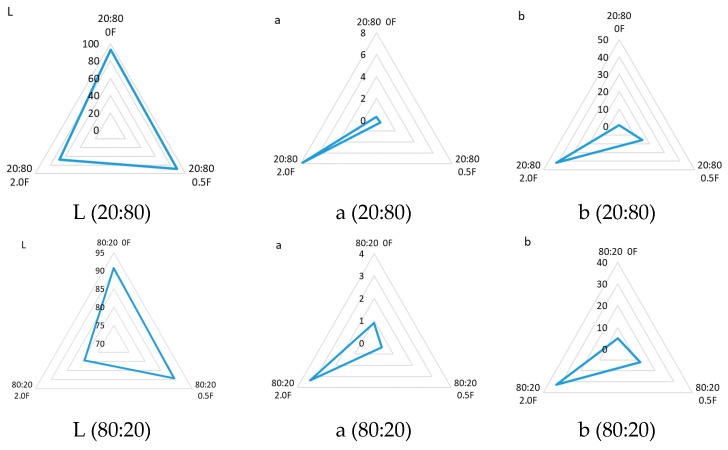
Results of color parameters (*L, *a, *b) obtained for pectin films.

**Figure 9:**
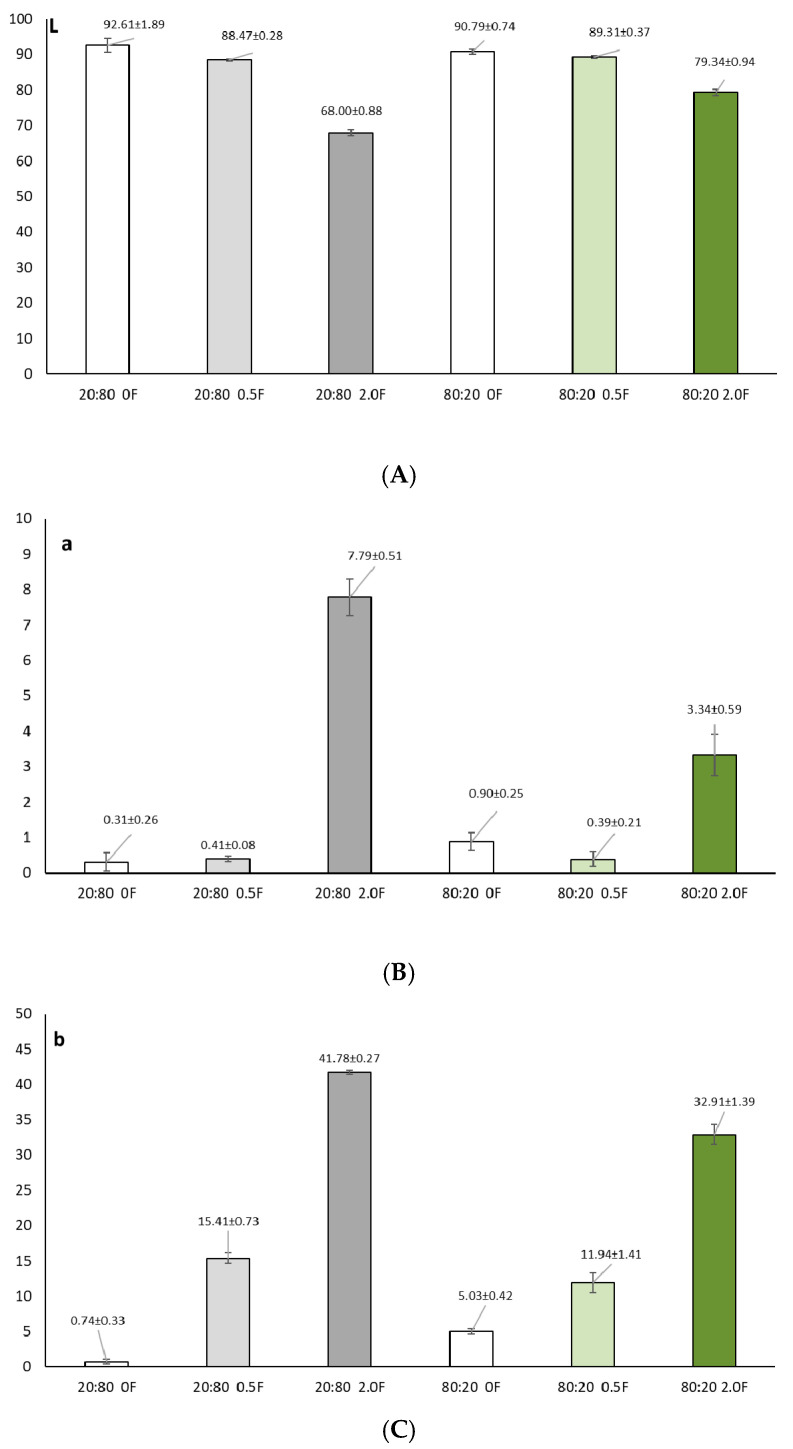
Color measurement results (L* (**A**), a* (**B**), b* (**C**)) of antioxidant pectin films (*p* > 0.05).

Additional extract to the pectin matrices caused a decrease in the L* (brightness) parameter for all films tested. As the amount of cannabis herb extract increased, the brightness value was significantly lower (*p* < 0.05). Samples 0, i.e., those without the extract, were characterized by values at 20:800F L* = 92.60 ± 1.89 and 80:200F L* = 90.78 ± 0.73. Figure 8 presents diagrams of the color parameters for all antioxidant pectin films. At the same time, an increase in the parameters a* (share of red color) and b* (share of yellow color) was observed in all films. This was related to the amount of polyphenols in the tested films. As the content of polyphenols increased, a statistically significant (*p* < 0.05) decrease in brightness was observed, as was an increase in the red and yellow color of the tested films (Figure 9). This effect was caused by the presence of active and color compounds in the extract, which, dispersed in the biopolymer matrix, changed the optical properties of the material [69,70]. The values obtained from the color evaluation were confirmed by studies performed with a stereoscopic microscope, on which the color of the tested samples could be clearly seen. The results of the color and correlation analyses were confirmed by Spearman’ rank correlation analysis. The strongest (and statistically significant) correlations suggested a significant effect of polyphenol content on the color, transparency and antioxidant properties of the samples.

### 3.7. Images of Antioxidant Pectin Films

**Figure 10:**
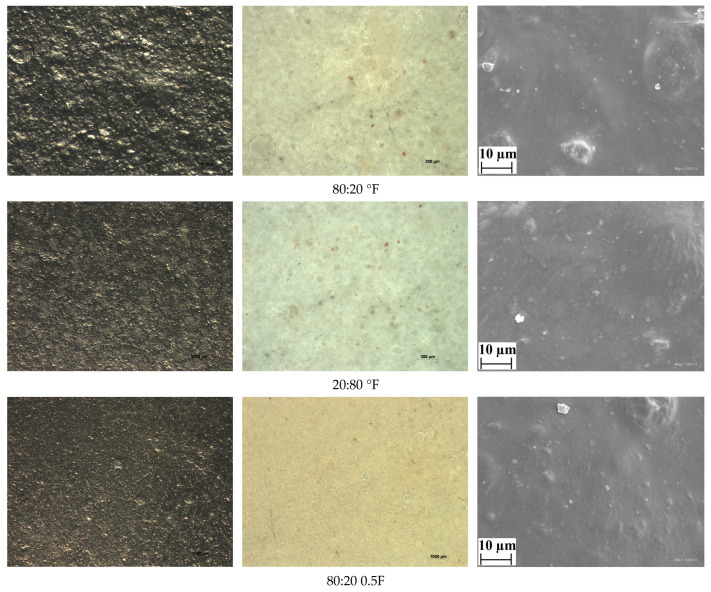

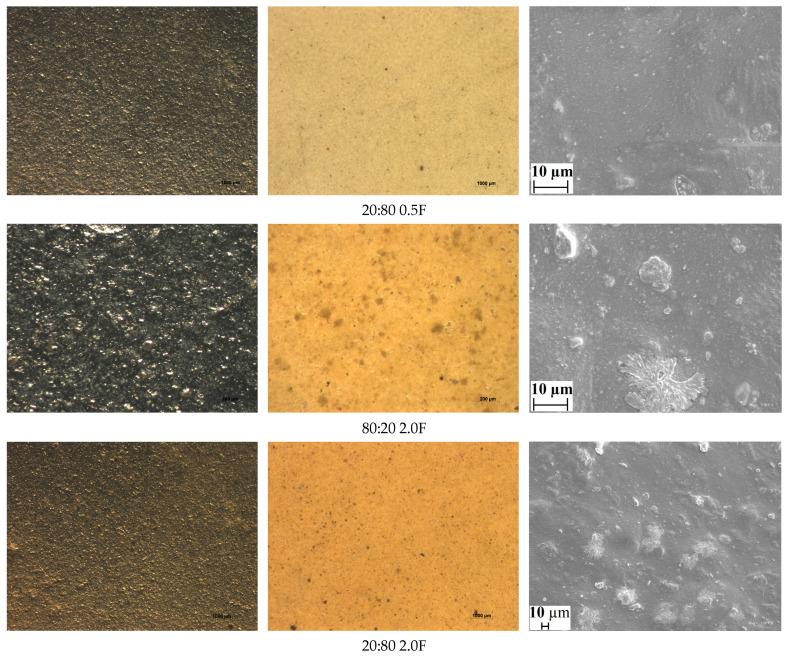
SEM and stereoscopic images for antioxidant pectin films. Scale bar: 10 μm.

In this study, scanning electron microscopy (SEM) and stereomicroscopy were employed to evaluate the surface of the films. SEM images (scale bar: 10 μm) were used to examine the morphology and surface characteristics of the obtained films (Figure 10). All samples analyzed by SEM showed smooth, crack-free surfaces without any evidence of phase separation, indicating consistent and strong interactions within the polymer matrix. The incorporation of pectin and polysaccharide extracts at appropriate concentrations resulted in dense and uniform surfaces, which is in agreement with the mechanical and barrier properties obtained. Similar findings were reported in previous studies [71], where starch-based films also exhibited homogeneous structures without cracks, pores, or phase separation. The stereomicroscopic analysis conducted in this work confirmed the smoothness observed in the SEM images and additionally revealed a clear change in film color depending on extract concentration, which is consistent with the results of the colorimetric analysis.

### 3.8. Statistical Analyses the Results

**Figure 11:**
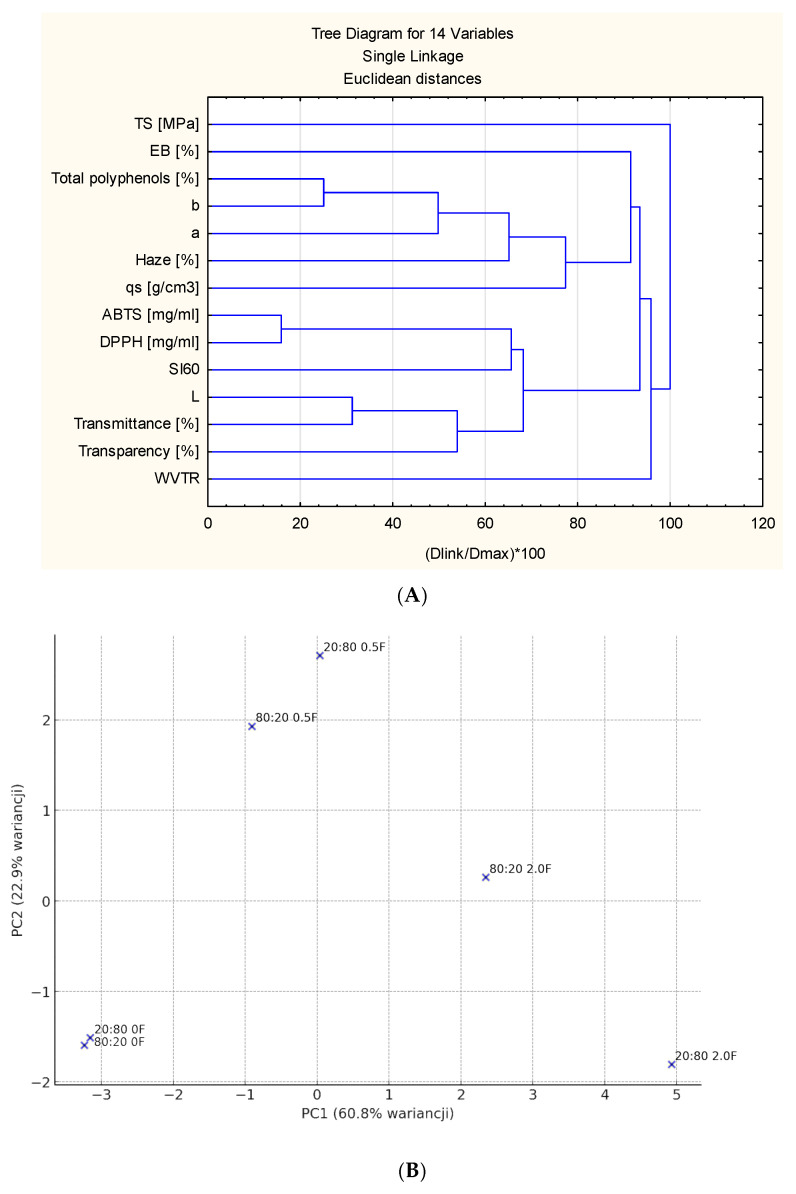
(**A**) Cluster analysis of the determined parameters of the studied films (*p* < 0.05); (**B**) PCA analysis.

Based on the results obtained, a cluster analysis, i.e., clustering of variables, was performed (Figure 11). The purpose of this was to identify variables that were comparable to each other in terms of how they differentiated the data (i.e., variables that co-occurred in a similar way). The characterization variables for antioxidant activity, particularly IC50, as determined by the DPPH radical and ABTS assays, were found to be highly correlated. The color components in the CIELAB color space were related to the content of total polyphenols, which is typical, since polyphenols often impart intense color to plant extracts or biodegradable plastics with natural additives. Transmittance and transparency, as well as the *L parameter (color brightness), represent variables associated with the external appearance or visual properties of the material. Therefore, they can be considered a separate group related to appearance and color. In addition, the swelling index (Si60) can serve as an indicator of surface or structural characteristics. The water vapor transmission rate (WVTR), on the other hand, was independent of optical, chemical, or mechanical properties and thus represented a different category of characteristics, namely the moisture barrier. The mechanical variables TS (tensile strength) and EB (elongation at break) were clustered together, but relatively late compared to in other groups. Although they belong to the category of mechanical properties, they differed in the context of the other variables and may indicate different mechanisms of material deformation. Finally, turbidity and density were clustered together but separated from strictly optical properties (transmittance and transparency). This may suggest that turbidity and density share a common source (e.g., light scattering by fillers or particles), although they are not typical “optical” variables. The PCA plot (Figure 11B) shows how the samples were distributed according to their first two principal components (PC1 and PC2), which together explain a substantial portion of the total variance in the dataset. We can observe that samples with similar extract content tend to group closer together in the PCA space, suggesting that this variable may be associated with certain shared characteristics captured by PC1 and PC2. The clear separation between some groups along PC1 or PC2 indicates that the underlying measured properties (such as mechanical, optical, or chemical parameters) differ significantly between formulations. In summary, PCA not only reduces the dimensionality of the dataset but also reveals meaningful trends, highlighting the influence of extract content on sample similarity.

**Figure 12:**
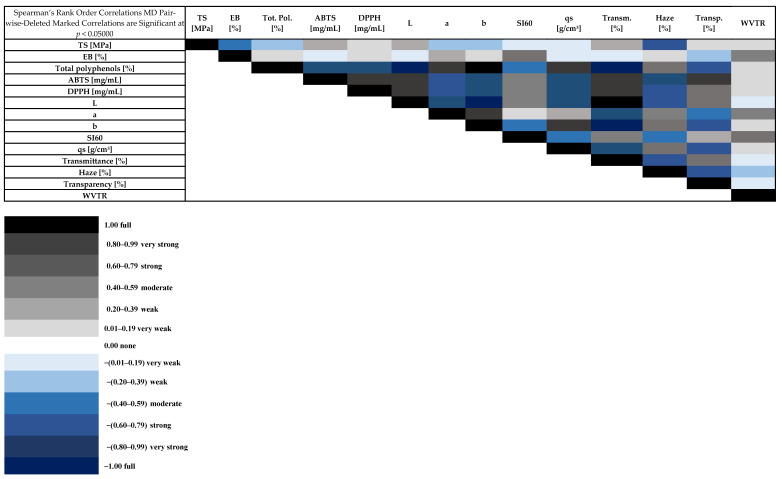
Spearman’s rank order correlations (*p* < 0.05000).

The Spearman’s rank correlation coefficients (Figure 12) describe the magnitude and direction of the relationships between pairs of variables. High positive correlations were present between the free radical neutralizing capacity of the ABTS and DPPH tests. The strong negative correlation between total polyphenols vs. ABTS and total polyphenols vs. DPPH was due to the inverse interpretation of the results in these tests (i.e., lower value = better activity). There was a strong positive correlation between density (qs) and total polyphenols. The brightness of the sample was positively correlated with antioxidant activity. Spearman analysis allowed us to conclude that at higher polyphenol contents, light transmittance decreased significantly (film samples became more opaque). The more flexible samples had a higher SI60 index. This is evidenced by the high correlation between the EB (elongation at break) and SI60 ratio. More flexible films allowed more water vapor to pass through. In summary, there were strong correlations between antioxidant indices and polyphenol content. Optical (transparency; haze) and mechanical (TS; EB) properties were also related to polyphenol content and antioxidant activity. The strongest (and statistically significant) correlations suggested that polyphenol content strongly influenced the color, transparency and antioxidant properties of the samples. They showed that WVTR was independent of optical, chemical or mechanical properties.

## 4. Conclusions

Biopolymer-based packaging materials are a promising alternative to conventional packaging due to their inherent biodegradability, non-toxicity, and biocompatibility. Additionally, they can incorporate antioxidant agents that effectively slow down undesirable biological changes in packaged foods, responding to the growing demand for active packaging systems and the need for safe products with extended shelf life. The incorporation of plant extracts into the polymer matrices significantly increased both total polyphenol content (TPC) and antioxidant activity in the films. The samples with the highest extract content (20:80 2.0F and 80:20 2.0F) exhibited a TPC of 0.9067 ± 0.0184 and the highest antioxidant activities (ABTS = 1.79 ± 0.04; DPPH = 4.44 ± 0.10). Mechanical testing revealed that films without extract, regardless of the PA to PC ratio, showed similar tensile strength (TS) and elongation at break (EB) values (*p* > 0.05). The addition of extract, and consequently the increase in polyphenol content, led to higher strength and reduced elasticity in the 80:20 films. Optical properties were also affected by the extract: all films showed decreased L* (brightness) values and increased a* (red component) and b* (yellow component) values. Spearman’s correlation analysis indicated that higher polyphenol content significantly reduced light transmittance, making the films more opaque, while more elastic films allowed greater water vapor transmission. Strong correlations were observed between antioxidant indices, polyphenol content, and both optical (transparency; haze) and mechanical (TS; EB) properties. These results demonstrate the potential of the developed films as active packaging materials. Natural antioxidants incorporated into polymer matrices can scavenge free radicals and chelate metal ions, thereby delaying oxidation and extending food shelf life. It should be noted that antimicrobial activity was not assessed in this study, which limits the full characterization of the materials. Future work will include antimicrobial assays to complement these findings and expand the potential applications of the films.

## Figures and Tables

**Table 1:** GC-MS of cannabis herb extract.

Peak RT (min)	Area	Quantity (%)	Compound Name	Formula	Prob. (%)	Main Characteristic *m*/*z*
9.545	4.60 × 10^6^	0.492	Cyclohexanecarboxylic acid, 2-hydroxy-, ethyl ester	C_9_H_16_O_3_	14.1	55, 73, 101, 127, 144, 207, 267
10.877	2.31 × 10^6^	0.247	6-Methylenebicyclo[3.2.0]hept-3-en-2-one	C_8_H_8_O	12.4	51, 65, 91, 120, 207
10.901	2.34 × 10^6^	0.25	Coumaran	C_8_H_8_O	18.6	65, 91, 120, 207
13.500	2.66 × 10^6^	0.284	cis-5,8,11,14,17-Eicosapentaenoic acid	C_20_H_30_O_2_	5.72	67, 79, 91, 133, 161, 207, 267, 341, 391
13.966	2.96 × 10^6^	0.316	10-Heptadecen-8-ynoic acid, methyl ester, (E)-	C_18_H_30_O_2_	16.3	57, 73, 93, 121, 281
15.552	6.21 × 10^6^	0.665	Caryophyllene oxide	C_15_H_24_O	12.0	55, 91, 192, 281, 327
15.877	2.02 × 10^6^	0.216	5,8,11,14-Eicosatetraenoic acid, methyl ester, (all-Z)-	C_21_H_34_O_2_	6.65	67, 81, 96, 192, 281, 327
15.985	3.76 × 10^6^	0.402	Cyclopenta[1,3]cyclopropa[1,2]cyclohepten-3(3aH)-one, 1,2,3b,6,7,8-hexahydro-6,6-dimethyl-	C_13_H_18_O	19.7	55, 77, 133, 175, 281
18.151	2.48 × 10^7^	2.657	Ethanol, 2-(9-octadecenyloxy)-, (Z)-	C_20_H_40_O_2_	10.8	55, 68, 82, 123, 207,263, 306
18.21	5.14 × 10^6^	0.55	2-Hexadecanol	C_16_H_34_O	12.8	55, 207, 280, 341
20.722	3.56 × 10^6^	0.381	6,9,12,15-Docosatetraenoic acid, methyl ester	C_23_H_38_O_2_	7.72	55, 67, 93, 149, 207, 281, 355
21.893	7.82 × 10^6^	0.836	Gitoxigenin	C_23_H_34_O_5_	37.7	55, 91, 115, 203, 221, 243, 286
21.999	2.93 × 10^6^	0.313	9,12,15-Octadecatrienoic acid, 2,3-dihydroxypropyl ester, (Z,Z,Z)-	C_21_H_36_O_4_	11.6	67, 129, 207, 229, 279, 355, 401
23.046	9.37 × 10^6^	1.002	Dihydrovallesiachotamine	C_21_H_24_N_2_O_3_	14.1	59, 129, 207, 281, 327, 375
23.561	7.55 × 10^8^	80.78	Cannabidiol	C_21_H_30_O_2_	52.1	55, 67, 91, 115, 147, 174, 207, 231, 246, 258, 314
24.625	7.12 × 10^7^	7.610	Cannabigerol	C_21_H_32_O_2_	54.4	67, 123, 193, 231, 281, 316, 341, 387

**Table 2:** Results for density of pectin films.

Samples Films	Density
	ρs [g/cm^3^]
20:80 °F	0.9340 ± 0.0125
20:80 0.5F	1.0001 ± 0.0115
20:80 2.0F	1.0021 ± 0.0109
80:20 °F	0.9699 ± 0.0394
80:20 0.5F	0.9951 ± 0.0373
80:20 2.0F	1.0048 ± 0.0096

## Data Availability

The original contributions presented in this study are included in the article. Further inquiries can be directed to the corresponding authors.
